# LATER-ADULTHOOD TRAUMA RE-ENGAGEMENT AMONG VIETNAM VETERANS: A COORDINATED ANALYSIS

**DOI:** 10.1093/geroni/igad104.0162

**Published:** 2023-12-21

**Authors:** Avron Spiro, Anica Pless Kaiser, Brian Smith, Jeanne Stellman, Steven Stellman

**Affiliations:** VA Boston Healthcare System, Boston, Massachusetts, United States; VA Boston Medical Center, Boston, Massachusetts, United States; Women’s Health Sciences Division, National Center for PTSD, Boston, Massachusetts, United States; Columbia University Mailman School of Public Health, New York City, New York, United States; Columbia University Mailman School of Public Health, New York City, New York, United States

## Abstract

Later-Adulthood Trauma Reengagement (LATR) is a framework for understanding the later-life emergence of trauma-related memories among aging veterans. LATR unfolds within the context of aging-related challenges such as retirement, physical decline, and bereavement. We developed a scale to assess LATR, the late-onset stress symptomatology short form (LOSS-SF). In this study, we compare the LOSS-SF scale across multiple samples of Vietnam veterans. Samples were surveyed between 2001 and 2020; all completed the LOSS-SF scale as well as measures of combat exposure and PTSD. We studied three male samples (256 repatriated US prisoners of war [RPW], 260 combat veterans [CV], and 507 deployed American Legion [AL] members) and one sample of 1956 women deployed to Vietnam, mostly nurses [WV]. Across samples, we examined three questions: 1) How prevalent is LATR? 2) Is LATR correlated with combat exposure and PTSD? 3) Using a regression framework, what factors (military characteristics, combat, PTSD) are related to LATR? Prevalence of LATR ranged from 5% to 38%. Mean LATR scores were higher among AL and CV samples than among the RPW and WV. LATR was modestly related to combat exposure (rs = .08 - .36) and moderately related to current PTSD symptoms (rs = .56 - .79). Regressions demonstrated the importance of PTSD to LATR across samples, and combat exposure was related to LATR only for WV. This study indicates that LATR varies across samples differing in gender and warzone experience and suggest further exploration of the overlap between PTSD and LATR among aging Vietnam veterans.

